# Disease advocacy organizations catalyze translational research

**DOI:** 10.3389/fgene.2013.00101

**Published:** 2013-06-04

**Authors:** Sharon F. Terry

**Affiliations:** ^1^Genetic AllianceWashington, DC, USA; ^2^PXE InternationalWashington, DC, USA

**Keywords:** rare diseases, advocacy, ABCC6, PXE, open access

## Abstract

Disease advocacy organizations have long played an important role in the continuum from basic science to therapy development in rare disease research. PXE International has led the field in innovative ways, venturing into specific activities that have traditionally been conducted by scientists. As lay founders, we have engaged in gene discovery, gene patenting, diagnostic test development, epidemiological studies, clinical trials, and therapy research and development. This article will describe the steps that we took, and the ways in which we have scaled these efforts for the larger community.

## ONE DISEASE

This perspective is that of individuals, families, and communities engaging in the scientific process to accelerate and improve health. We are ordinary parents, like hundreds working to better the lives of their children. Our original focus on our children’s disease has become agnostic to disease, and expanded to include broad systemic change in the clinical and translational research enterprise.

Our quest began in 1993, when we noticed some small lesions on the sides of our 7-year-old daughter Elizabeth’s neck. After a year of the diagnostic odyssey, we took her, out of plan and out of pocket, to a dermatologist, Lionel Bercovitch, MD, who recognized pseudoxanthoma elasticum (PXE) immediately. Looking at Elizabeth’s 5-year-old brother, Ian’s neck, and said, “He has it too.” Then he examined Elizabeth’s eyes. He was the perfect diagnostician for this condition; he was also trained as an ophthalmologist. Before this experience, we had no idea that a skin disease could be systemic. We had no frame of reference for all of this foreign information: “systemic, genetic, recessive, papules, angioid streaks…”

Our response, besides showering our children with gifts that Christmas 1994, in the pre-internet age, was to photocopy every article we could find on the disease: a stack of about 400 articles. We could not understand them and so turned to medical dictionaries and reference material.

By the middle of January, we understood several important things: (1) no one knew how this disease progressed, there were conflicting conclusions in the papers we read, (2) there was no comprehensive plan to study the disease, nor was there a plan emerging, (3) no one even knew how many people had the disease, and (4) there was no treatment, the gene had not even been discovered yet!

In the midst of this morass, two researchers from two different prominent biomedical research institutions appeared. After the first took blood from all of us, the second wanted the same. We told him to go get some from the first one. The chuckled and we learned the astounding fact that that scientists competed: they did not collaborate.

**FIGURE 1:**
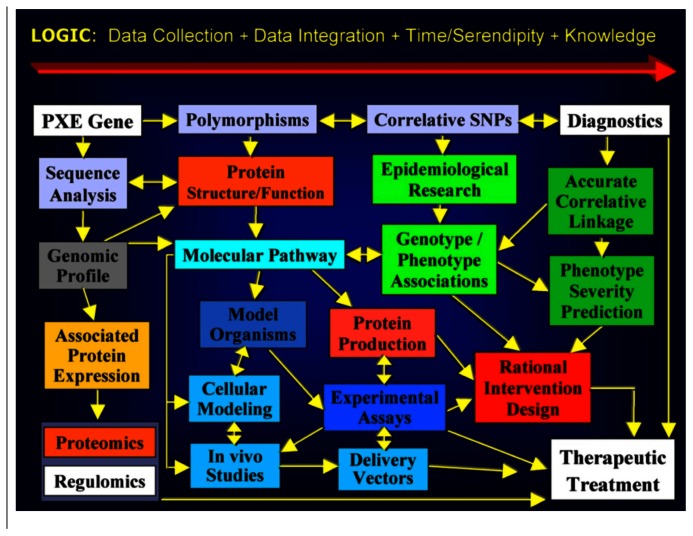
**PXE International’s 1995 Strategic Plan (published in Terry and Boyd, 2001)**.

Within a few months of the start of our informal education in PXE, genetics, dermatology, ophthalmology, cardiology, biomedical research, and therapy development, we devised a plan largely influenced by Patrick’s background in building engineering. **Figure 1** shows the “wiring diagram” plan for advancing research to lead to interventions (Terry and Boyd, 2001).

We first enlisted Dr. Bercovitch, asking him to be medical director and board member of a foundation we named PXE International. Then we asked the nearest lab engaged in the search for the PXE gene, if we could wash test tubes to speed up their research. They generously allowed us to come into the lab in the evenings and eventually gave us keys. They did not want us washing test tubes, they wanted us to “score gels.” And so we scored gels night after night. Patrick often stayed until the wee hours of the morning. We had a wonderful neighbor who would watch our kids in the evenings while they slept.

Simultaneous with this we started to build a cohort of well-characterized individuals affected by PXE. We contacted dermatologists and ophthalmologists around the world and started adding people to our registry. We held meetings in Boston, New York, California, Paris, Gent, Modena, Amsterdam, and Cape Town. We used the nascent internet and created listservs.

We also contacted all of the researchers who had written numerous papers on PXE. We asked if we could meet with them, and to a person, they were generous and open with their time. Ken Neldner (Neldner, 1988), Mark Lebwohl (Lebwohl et al., 1994), Jouni Uitto (Christiano et al., 1992; Christiano and Uitto, 1994), Anne De Paepe (Godfrey et al., 1995), Ivonne Ronchetti (Contri et al., 1996), Charles Boyd (Lebwohl et al., 1994), Arthur Bergen (van Soest et al., 1997), Michael Pope (Pope, 1975), and Dennis Viljoen (Viljoen, 1988), all met with us in their labs, gave us tours and educated us.

We recommended to the researchers that they all work together to find the gene. People told us “you can’t herd cats, so stop trying.” We retorted, “yes you can, you just need to move the food.” We set to work building the first ever lay-owned blood and tissue bank (Terry et al., 2007; Terry, 2008). We collected blood samples by sending kits to affected individuals all over the world. With epidemiologists, we created a survey instrument and administered it, including collecting pedigrees. We then gave researchers access to the de-identified samples and data if they agreed to play by our then novel data sharing rules.

We had some sophisticated help in those days. Having met Francis Collins, then director of the National Human Genome Research Institute (NHGRI) at the National Institutes of Health, at the 10th anniversary of the Alliance of Genetic Support Groups (now known as Genetic Alliance), we asked for advice and he shared NHGRI’s technology transfer wizard with us. Claire Driscoll helped us craft state of the art consents, protocols, material transfer agreements, and then joined our board upon which she still serves. The work she did formed the underpinning of our later cross-disease efforts.

We were not able to get all research groups to share data in those early days, but we did get a few to combine forces resulting in back-to-back papers in Nature Genetics (Bergen et al., 2000; Le Saux et al., 2000) when the gene was discovered through our wet bench work and that of several other groups (Ringpfeil et al., 2000). We were also able to encourage focus on the discovery of the gene associated with PXE, despite the attractiveness of several unknown genes in the locus. This is evidence of the contributions communities can make even in basic research related to a disease.

Our work moved from scoring gels to entering and analyzing the data, meeting with the various teams around the world search for the gene, and materially participating in the discovery. With a group of about five scientists we discovered the: one of the known ones-ABCC6. This discovery taught us a very important lesson. It was thought that the gene would code for a protein involved in a structural aspect of elastin, since degredated elastin fibers are common in all of the organs affected by PXE. Instead, ABCC6 codes for a membrane transport protein, in the same family as cystic fibrosis. The big learning for us, which we encounter over and over in biology, is that we do not know which discoveries are going to benefit one disease or another. We often quote: “a rising tide lifts all boats.” This experience was critical to our thinking about this disease and others on a system level. It later informed a policy position Genetic Alliance took about not earmarking federal funds for specific diseases (Terry, 2010).

We are co-inventors and patent holders of ABCC6 with the other scientists. We have assigned our rights to PXE International and as such are stewards of the gene, making sure there is open access to it for research and therapeutic development.

## ALL DISEASES

Throughout these years, we frequently met with individuals affected by PXE and their families around the world. We built a robust website at pxe.org and created volumes of information on the disease to help mitigate the diagnostic odyssey and lack of information.

From the beginning we had excellent mentors in disease advocacy organizations. We also had hundreds of requests to help other organizations set up registries, biobanks, and research enterprises. I moved my work to a dynamic umbrella organization called Genetic Alliance, and was joined by remarkable colleagues who also sought the most effective systems-level solutions to accelerate translational research and services for all. Together we created a collaborative network that has led to the development of many tools, resources, and even legislation (**Table 1**).

**Table 1:** Resources and tools to accelerate research and services.

Need	Tool or resource	Year	Reference
Cross-disease, common platform, biobank, and registry	Genetic Alliance Registry and BioBank (www.biobank.org)	2003	Landy et al. (2012)
Toolbox/manual for maintaining an advocacy organization	WikiAdvocacy (www.wikiadvocacy.org)	2004	Weiss (2004)
Disease information provided by the experts (disease advocacy organizations) and filtered from federal sources to be at the right literacy level	Disease InfoSearch (www.diseaseinfosearch.org)	2006, revised in 2013	Landy et al. (2012)
The power of family history	Does It Run in the Family? (www.familyhealthhistory.org)	2006	O’Leary et al. (2011)
Protection against discrimination based on genetics	Coalition for Genetic Fairness and passage of the Genetic Information Nondiscrimination Act of 2008 (www.ginahelp.org)	2008	Dressler and Terry (2009); Terry (2009)
Clarity about the reliability of health information on the internet	Trust It or Trash It (www.trustortrash.org)	2009	NA
Information about newborn screening in all 50 states	Baby’s First Test (www.babysfirsttest.org) as a result of the Newborn Screening Saves Lives Act of 2008	2010	NA
Drug development seen as a network, rather than a pipeline	Navigating the Ecosystem of Translational Science (www.geneticalliance.org/nets)	2011	Baxter et al. (2013)
Cross-disease registries for all that allow the individual to set their sharing and data access settings	Registries for All (www.reg4all.org)	2013	Terry et al. (2013)
Clinical trials need to find the individual, not the other way around	TrialsFinder (www.trialsfinder.org)	2013	NA
Resources for the public to understand genetic technologies	Genes In Life (www.genesinlife.org)	2013	NA

## CULTURE CHANGE

Changes taking place in society in the areas of information technology and networks, if parlayed for improving health, will be an essential catalyst for the transformation of biomedical research. The current biomedical research system was modeled after an industrial age culture of scarcity, win–lose, linear progress, and competition. We live in an age where raw materials are abundant: information is being produced today at rates we cannot manage. A newborn has more information shared about her in the first days of life than the US Library of Congress contains. Networks, concurrent processes, win–win engagements, and ever increasing transparency and openness are now available to transform the research enterprise.

These changes have initiated a wonderful rebirth in systems surrounding non-profits such as Genetic Alliance. It is critical for us to work with other organizations in a boundary-less way. This requires that we ask “how is it true of me?” when we encounter an obstacle that appears to be external. This is our practice both as individuals in Genetic Alliance and PXE International and for the organizations themselves. It is our belief that each of us is responsible because we not only represent the whole: we are the whole. It is also critical for us to be ever vigilant of the downfall of all systems: that they begin to exist to largely to protect the system rather than to serve the mission (Meadows, 2008). Just as we each look in the mirror every day and ask, “Am I the best person for this job?,” it is critical we ask if Genetic Alliance or PXE International are the best organization for their respective missions.

## THE FUTURE

Advocacy organizations of the future will not look like today’s organizations that were built on models such as Alcoholics Anonymous. Today’s young parents do not join one group, one organization. They join many affinity groups and are adept at managing them. They create custom solutions that meet their family’s needs. They use multiple ways of interacting, without compartmentalizing their lives. They do not experience the same level of isolation based on their children’s diagnosis as we did in 1994. Parents today do not identify with one aspect of life to the detriment of others.

It’s never wise to predict the future. I do believe, however, that we need to be bold in our vision of the future. I think that if we do not risk it all, and lead to the highest place we can envision, we will not succeed in our lofty, and essential, goals. I believe that we need to work together, without regard for the histories of our organizations, or body geography of the diseases for which we seek to find therapies.

Tools to help us achieve grand challenges have emerged. Data sharing in the information age is transformative – it will break down barriers and accelerate translational and clinical science. Giving individuals and communities the tools to decide with whom to share their data and samples, and how much to share is essential. When we understand that our fear of sharing information is hugely detrimental to accelerating solutions we will free up a great deal of energy. The old system will not work. Advocacy organizations, academic institutions, companies, and legislators still cling to it since it is familiar and safe. It is hard for us to see how unsafe it is to remain in the old models, and that it will impede our efforts. We have ample examples in other industries: music, travel, and publishing. Consumers have effected that change. As consumers in the cottage industry we call healthcare we are disconnected from our needs, and cannot feel them in the same way as we feel the need for music or air travel options and accessibility. This is remarkable because so much is at stake. Special interests, uncoordinated systems, lack of evidence, a “non-learning” healthcare system and fear keep us from achieving better health for all.

The advocacy organizations of the future will be flexible and dynamic. Their boards of directors will not focus on sustaining the organization; they will focus on maximizing the advance to the goals. They will be cross-disease, and be constellated around biological pathways, phenotypes, and biomarkers. They will come into being to address a very critical problem and dissolve or move on once that problem is addressed. The advocacy organization of the future will be an integral part of the research enterprise and not so novel in its work that it would be worthy of this sort of paper.

It is time to align incentives to serve the millions around the globe who suffer. It is time to risk what we think is unthinkable, share information and be bold. There is no time to hesitate – our loved ones cannot wait.

## Conflict of Interest Statement

The author declares that the research was conducted in the absence of any commercial or financial relationships that could be construed as a potential conflict of interest.
